# Post-traumatic tension faecopneumothorax in a young male: case report

**DOI:** 10.1186/1749-7922-3-20

**Published:** 2008-07-12

**Authors:** J Kelly, ET Condon, WO Kirwan, HP Redmond

**Affiliations:** 1 Department of General Surgery, Cork University Hospital, Wilton, Cork, Ireland

## Abstract

Diaphragmatic rupture due to trauma is both well recognised and uncommon. The difficulties in diagnosing traumatic diaphragmatic rupture at the first admission are the most common causes of latent morbidity and mortality. Herniation of the abdominal viscera is the most common sequel with strangulation and perforation the most serious complication. This case outlines the delayed presentation of diaphragmatic rupture and herniation presenting as an acute tension faecopneumothorax. We review the relevant literature, with particular emphasis on the difficulties in diagnosis at presentation.

## Case presentation

A 22 year old male presented to the Emergency Department having been stabbed with a six inch kitchen knife in the left costal margin during a violent assault. Clinically it was felt to be superficial. CT thorax, abdomen and pelvis failed to identify any breach of the thoracic or abdominal cavities. There was no free fluid/air and no evidence of hollow viscous injury. His wound was locally explored and he was admitted for overnight observation. He remained well and was discharged the following day.

**Figure 1:**
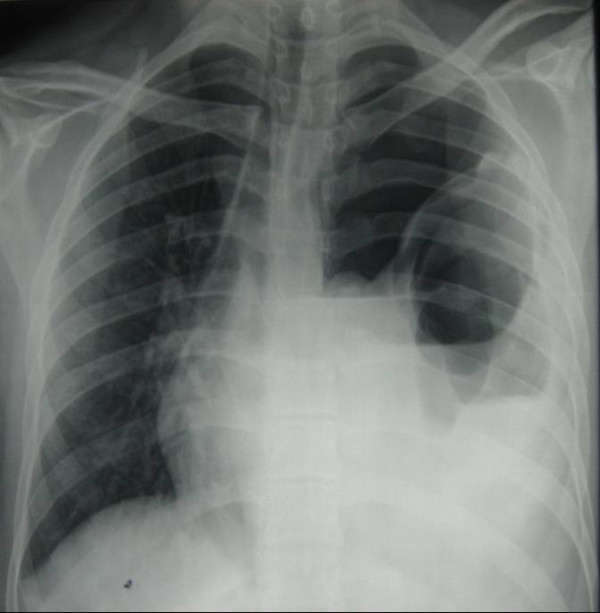
**chest radiograph shows large colon in the left hemi-thorax with an air-fluid level.** There is associated contralateral shift of the mediastinal contents.

**Figure 2:**
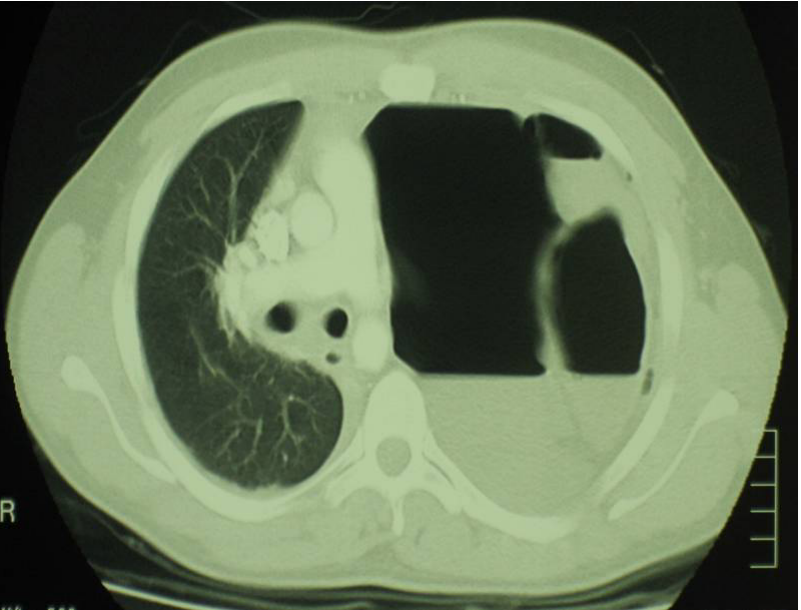
CT scan shows a left hydropneumothorax, total collapse of the left lung, contralateral shift of the mediastinal contents under tension and left anterior diaphragmatic rupture with herniated perforated transverse colon.

Six months later he presentated with an acute deterioration in his respiratory status. He was complaining of left sided chest pain and was dyspnoeic with saturations of 85% on room air. His BP was 90/60 and his pulse rate was 130 bpm. He was in obvious distress and had engorgement of his neck veins. He had no air entry on the left side and had some tenderness in the left upper quadrant without signs of peritonism. An erect chest x-ray revealed a loop of colon herniating through his left diaphragm into his hemi-thorax, with associated mediastinal shift (figure 1). CT later confirmed tension hydropneumothorax (figure 2). A thoracostomy tube was inserted and 700 mls of faeculent fluid emerged.

An immediate thoracolaparotomy (anterior left side) was performed and showed a strangulated, gangrenous left sided diaphragmatic hernia containing a perforated loop of transverse colon. The transverse colon was resected. A Paul/Mikulicz type colostomy was performed and primary repair of the left diaphragm with 1/0 vicryl continuous suture. He remained in the intensive care unit for 8 days. He made an otherwise uneventful recovery. His colon was reanastomosed six months later and he remains well.

## Discussion

Acquired traumatic diaphragmatic hernia is a well-known complication following both blunt and penetrating injuries to the diaphragm, occurring in 5% of those who experience major blunt trauma. 95% of ruptures are on the left side because of the protective nature of the liver on the right. Subsequently right sided rupture is associated with higher rates of morbidity and mortality.

Diaphragmatic rupture is frequently not recognized at the time of trauma because of the non-specific, varied and confusing clinical signs and radiographic findings. The interval between injury and the onset of symptoms can range from several weeks to years. In some cases the delay in diagnosis is due to absence of symptoms at the time of injury. The initial absence of symptoms at the time of injury may be due to absence of associated herniation or prolapse of intra-abdominal viscera into the chest cavity when the diaphragmatic rupture occurred. Some of the patients only become symptomatic when there is complication to herniated organs, such as obstruction, strangulation or perforation. When the presentation is late the diagnosis may be difficult because the causal association to the original injury isn't always clear [1]. Degiannis et al [2] reported, in their retrospective series of 45 patients with diaphragmatic hernia post penetrating trauma, that 16/45 patients had a delayed diagnosis (median of 27 months). The mortality rate in this group was 25% compared to 3% in the group who presented early.

Chest radiograph is the most important diagnostic image and may show elevation of the hemidiaphragm, a bowel pattern in the chest, or an NG tube passing into the abdomen and then curling up into the chest. However chest x-ray is only suggestive in 17 – 48% of the cases [3]. On CT scans, herniation of an organ or omental fat may be visible through an abrupt discontinuity in the diaphragm. A waistlike constriction (collar sign) produced by diaphragmatic compression of herniated organs may be seen.

A cautious approach must be taken with diagnostic laparotomy when the suspicion of traumatic diaphragmatic injury emerges because of the potential of the pneumoperitoneum leaking into the chest cavity and subsequently causing an iatrogenic tension pneumothorax.

Recent studies have shown some benefit of diagnostic laparoscopy in the detection of intra-abdominal injuries including injuries to the diaphragm, but only in the setting of haemodynamically stable patient [4,5].

## Conclusion

"Tension faecopneumothorax" is a rare but serious complication following post-traumatic diaphragmatic hernia from a penetrating stab wound to the chest. Surgeons should have a high index of suspicion for abdominal visceral injuries in the acute presentation of these injuries. Laparoscopy may be beneficial in some cases. Delays in diagnosis are the main causative reason for excessive morbidity and mortality.

## Consent

Written informed consent was obtained from the patient for publication of this case report and any accompanying images. A copy of the written consent is available for review by the Editor-in-Chief of this journal.

## Competing interests

The authors declare that they have no competing interests.

## Authors' contributions

JK and ETC conceived of the study, carried out a detailed literature review, collected and presented the pertinent data. WOK and HPR participated in the study design and coordination and helped to draft the final manuscript. All authors read and approved the final manuscript.

## References

[B1] Vermillion JM, Wilson EB, Smith RW (2001). Traumatic diaphragmatic hernia presenting as a tension fecopneumothorax. Hernia.

[B2] Degiannis E, Levy RD, Sofianos C (1996). Diaphragmatic herniation after penetrating trauma. Br J Surg.

[B3] Ziehren J, Enzweiler  C, Müller JM (1999). Tube thoracostomy complicates unrecognized diaphragmatic rupture. Thorac Cardiovasc Surg.

[B4] Ivatury RR, Simon RJ, Stahl WM (1993). A critical evaluation of laparoscopy in penetrating abdominal trauma. J Trauma.

[B5] Ortega AE, Tang E, Froes ET (1996). Laparoscopic evaluation of penetrating thoracoabdominal traumatic injuries. Surg Endosc.

